# High Performance Graphene–C_60_–Bismuth Telluride–C_60_–Graphene Nanometer Thin Film Phototransistor with Adjustable Positive and Negative Responses

**DOI:** 10.1002/advs.202206997

**Published:** 2023-02-07

**Authors:** Rui Pan, Yuanlingyun Cai, Feifei Zhang, Si Wang, Lianwei Chen, Xingdong Feng, Yingli Ha, Renyan Zhang, Mingbo Pu, Xiong Li, Xiaoliang Ma, Xiangang Luo

**Affiliations:** ^1^ State Key Laboratory of Optical Technologies on Nano‐Fabrication and Micro‐Engineering Institute of Optics and Electronics Chinese Academy of Sciences Chengdu 610209 P. R. China; ^2^ Division of Frontier Science and Technology Institute of Optics and Electronics Chinese Academy of Sciences Chengdu 610209 P. R. China; ^3^ School of Optoelectronics University of Chinese Academy of Sciences Beijing 100049 P. R. China; ^4^ Research Center on Vector Optical Fields Institute of Optics and Electronics Chinese Academy of Sciences Chengdu 610209 P. R. China

**Keywords:** 2D material, bidirectional response, gate voltage regulation, graphene, phototransistor

## Abstract

Graphene is a promising candidate for the next‐generation infrared array image sensors at room temperature due to its high mobility, tunable energy band, wide band absorption, and compatibility with complementary metal oxide semiconductor process. However, it is difficult to simultaneously obtain ultrafast response time and ultrahigh responsivity, which limits the further improvement of graphene photoconductive devices. Here, a novel graphene/C_60_/bismuth telluride/C_60_/graphene vertical heterojunction phototransistor is proposed. The response spectral range covers 400–1800 nm; the responsivity peak is 10^6^ A W^−1^; and the peak detection rate and peak response speed reach 10^14^ Jones and 250 µs, respectively. In addition, the regulation of positive and negative photocurrents at a gate voltage is characterized and the ionization process in impurities of the designed phototransistor at a low temperature is analyzed. Tunable bidirectional response provides a new degree of freedom for phototransistors' signal resolution. The analysis of the dynamic change process of impurity energy level is conducted to improve the device's performance. From the perspective of manufacturing process, the ultrathin phototransistor (20–30 nm) is compatible with functional metasurface to realize wavelength or polarization selection, making it possible to achieve large‐scale production of integrated spectrometer or polarization imaging sensor by nanoimprinting process.

## Graphical abstract

Here, a novel graphene/C_60_/Bi_2_Te_3_/C_60_/graphene vertical heterojunction phototransistor is proposed. The transport process for photogenerated charge and local field mechanism are discussed in detail by analyzing the photoelectric performance of the device at low temperatures and different gate voltages. The device not only shows excellent photoelectric detection performance, but also exhibits substantive possibility to achieve large‐scale integrated array imaging sensors.

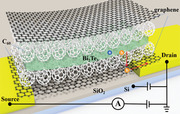

## 1 Introduction

As a representative of 2D materials, graphene is an important scientific research object due to its characteristics of high mobility, wide band absorption, adjustable energy band, etc.^[^ ^1^, ^2^, ^3^, ^4^, ^5^, ^6^ ^]^ These unique properties make it an ideal phototransistor material.^[^ ^7^, ^8^, ^9^ ^]^ Since Xia et al. prepared the first graphene phototransistor in 2009,^[^ ^10^ ^]^ graphene has been used in hybrid systems with various materials or even physical local fields to achieve photoelectric detection.^[^ ^11^ ^]^ For example, metasurfaces,^[^ ^12^, ^13^, ^14^ ^]^ quantum dots (QDs),^[^ ^15^ ^]^ nanowires,^[^ ^16^, ^17^ ^]^ bulk materials,^[^ ^18^ ^]^ transition metal dichalcogenide materials,^[^ ^19^, ^20^ ^]^ perovskite materials,^[^ ^21^, ^22^, ^23^ ^]^ and various organic substances^[^ ^24^, ^25^, ^26^ ^]^ have been combined with graphene to achieve a high performance photoelectric detection.^[^ ^27^ ^]^ These works have extensively promoted the research progress of graphene in the field of photoelectric detection. However, large‐scale array imaging is still a considerable challenge due to the engineering bottlenecks and theoretical limitations.^[^ ^28^ ^]^ To name a few, clean transfer of 2D materials and uniform growth of QDs/nanowires are the primary technical bottlenecks to achieve a homogeneous preparation of the array unit.^[^ ^29^ ^]^ Meanwhile, a high photoconductive gain is usually at the cost of sacrificing the response time due to the limitation of the defect state.^[^ ^30^ ^]^ Therefore, it is urgent to find a technical scheme that can realize the large‐scale uniform preparation of optoelectronic imaging devices with graphene and 2D materials.^[^ ^31^ ^]^

Herein, we propose a graphene/C_60_/bismuth telluride (Bi_2_Te_3_)/C_60_/graphene composite film phototransistor, which shows an excellent photoelectric response and strong technical and theoretical potentials for integrated photoelectric detection based on graphene. In the horizontal direction, graphene creates efficient and controllable conductive channels. In the vertical direction, the covalent contact of graphene/C_60_ forms a charge transport interface to the transmission and separation of the photogenerated charges.^[^ ^32^, ^33^ ^]^ The intermediate layer Bi_2_Te_3_ provides efficient light absorption in the near‐infrared band, broadening the response band of the device.^[^ ^34^ ^]^ At room temperature, this graphene/C_60_/Bi_2_Te_3_/C_60_/graphene phototransistor shows an ultrahigh peak responsivity (3 × 10^6^ A W^−1^), fast response (250 µs), and response band covering 400–1800 nm. Moreover, the device exhibits positive and negative photocurrent responses under different gate voltages, which result from the interaction between photogenerated electrons and dark electrons in the graphene channel. The graphene/C_60_/Bi_2_Te_3_/C_60_/graphene phototransistor not only presents a tremendous photoresponse performance but also shows excellent potential in array applications. The composite film can be combined with the functional metasurfaces^[^ ^35^, ^36^ ^]^ to form a single detector with polarization or wavelength selectivity, which makes it possible to realize spectrum detection and polarization imaging through array detector integration.^[^ ^37^ ^]^ Ultrathin thickness (30 nm) makes it possible to achieve a large‐scale production of integrated spectrometers or polarization imaging sensors by the nanoimprinting process.^[^ ^38^ ^]^

## 2 Results and Discussion

**Figure 1:**
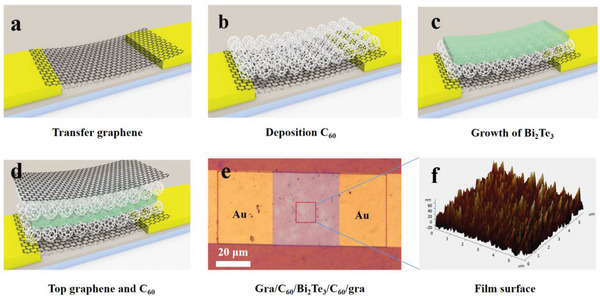
Fabrication process and structure morphology of the phototransistor. a) Single‐layer graphene is transferred to the substrate with prefabricated Au electrodes. b) Deposition of C_60_ on the single‐layer graphene. c) Growth of Bi_2_Te_3_ on C_60_. d) Deposit of C_60_ on Bi_2_Te_3_ and transfer of graphene. e) Optical microscope image of the obtained unit device. f) Atomic force microscope image of the composite films.

**Figure** **1** shows the schematic diagram of the device's preparation process and structure morphology. The metal electrodes (100 nm Au/10 nm Ti) were first deposited on the silicon oxide substrate by magnetron sputtering. A single layer of graphene was then transferred onto the substrate to cover the metal electrode using a wet transfer technique, as shown in Figure 1a. After a 5 nm C_60_ thin film was deposited by vacuum thermal evaporation at an evaporation temperature of 420 °C with a substrate temperature of 150 °C (Figure 1b), a layer of Bi_2_Te_3_ (20 nm) was subsequently deposited on C_60_ (Figure 1c). After that, another layer of C_60_ film (5 nm) and graphene was fabricated onto Bi_2_Te_3_ by the same process. Finally, the film was patterned by photolithography and etching process to complete the device preparation. Figure 1d is a schematic structural diagram of the device and Figure 1e displays the final device under the optical microscope. From the atomic force microscope (AFM), we can see that the thickness of the composite film is about 20–30 nm, as shown in Figure 1f. Figure S1 (Supporting Information) shows the optical microscope diagram of the device and AFM characterization details for the graphene/C_60_/Bi_2_Te_3_/C_60_/graphene film.

**Figure 2:**
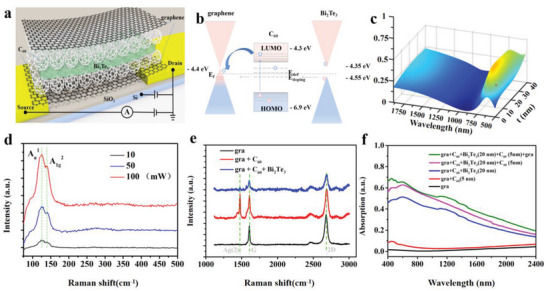
Characterization of graphene/C_60_/Bi_2_Te_3_/C_60_/graphene composite films. a) Schematic diagram of the electrical test of the unit device. b) The change of energy band structure at the graphene/C_60_/Bi_2_Te_3_ interface. c) The absorption spectra of the composite film as a function of Bi_2_Te_3_ thickness (*t*) calculated by FDTD simulation. d) Raman shift of Bi_2_Te_3_ film with a different incident light power of 532 nm. e) Raman shift for graphene (gra), graphene with C_60_, and graphene/C_60_/Bi_2_Te_3_. f) The experimental absorption of the composite film from 400 to 2400 nm.

As shown in **Figure** **2**a, the source–drain voltage is applied to the metal electrodes and the gate voltage is applied to the highly doped silicon during the electrical test. Figure 2b exhibits the change of energy band at the graphene/C_60_/Bi_2_Te_3_ interface and the localized energy levels in the bandgap caused by impurities and defects. As shown in Figure S2b (Supporting Information), a built‐in barrier preventing the hole injection into graphene is produced after the graphene is in contact with C_60_. As a result, the photogenerated electrons in C_60_ enter the P‐type graphene channel forming a negative photocurrent response with the visible light incident, as shown in Figure S2c,d (Supporting Information). Under the illumination of infrared light, the charges in C_60_ cannot be excited by the infrared photons of low energy due to band limitation. The Bi_2_Te_3_ film with narrow bandgap will respond to the infrared light and generated electron–hole pairs, but the barrier at the interface of C_60_ and Bi_2_Te_3_ limits electrons to enter C_60_. Since the valence band of Bi_2_Te_3_ is close to the impurity energy level in C_60,_ it provides a channel for photogenerated hole injection into P‐type graphene, and generates a positive photocurrent, as shown in Figure S2e (Supporting Information). Before preparing the composite film, we measured the refractive index of the Bi_2_Te_3_ thin film through the ellipsometer, as shown in Figure S3 (Supporting Information), and the relationship between the thickness of Bi_2_Te_3_ and the absorption spectrum of the composite film was obtained through finite difference time domain (FDTD) simulations, as illustrated in Figure 2c. Details on the simulation setup can be found in the Supporting Information. According to the calculated results, we can infer that the composite film shows the highest absorption peak when the thickness of Bi_2_Te_3_ is about 20 nm, which determines the structure in experiment. Figure 2d exhibits the Raman shift for Bi_2_Te_3_ film, the characteristic peak for Bi_2_Te_3_ can be clearly seen with different incident powers. Figure 2e presents the Raman shift for graphene, graphene with C_60_, and graphene/C_60_/Bi_2_Te_3_. The characteristic peaks of different materials appear subsequently, which proves the good quality of each film. The experimental absorption spectra of composite film in 400–1800 nm wave band are shown in Figure 2f, which is consistent with the simulated results in Figure 2c that a strong peak is located around 500 nm.

**Figure 3:**
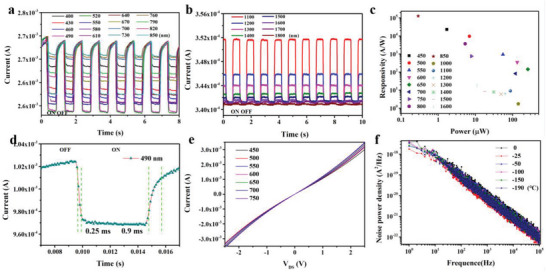
Photoelectric performance of graphene/C_60_/Bi_2_Te_3_/C_60_/graphene phototransistor. Photocurrent response in a) visible light band and b) in near‐infrared waveband. c) Responsivity for the device in the range of 400–1600 nm. d) Response speed of the device with incident light wavelength at 490 nm. e) *I*–*V* curve in the wavelength range of 450–750 nm. f) The noise power density of the device in different temperatures.

Next, we systematically measure the photoelectric performance of graphene/C_60_/Bi_2_Te_3_/C_60_/graphene phototransistor. **Figure** **3**a,b shows the device's photocurrent response in the visible and near‐infrared band at room temperature, respectively. And the photocurrent response in low temperature is presented in Figure S5 (Supporting Information). Furthermore, it can be seen that the photocurrent is negative in the visible range and positive in the near‐infrared range. To investigate the detection capability of the device at different wavelengths, we calculate the responsivity at different wavelengths, as shown in Figure 3c. The device exhibits an extremely high responsivity(1 × 10^5^ A W^−1^) at room temperature at 850 nm with a 0.3 µw incident light power. In addition, the phototransistor not only shows a high responsivity but also displays a fast speed response. As clearly presented in Figure 3d, the time of the falling edge is about 250 µs for the response time in a single waveform at 490 nm, and the rising edge is about 900 µs. In order to show the broadband response characteristics of the device in detail, we test the device's response time in the range of 400–850 nm, as shown in Figure S6 (Supporting Information). The *I*–*V* curve of the device is not a straight line, because the contact interface in the vertical direction forms a built‐in electric field, as shown in Figure 3e. The *I*–*V* curve of the infrared region is shown in Figure S4 (Supporting Information). Because the noise is directly related to the detection capability of the device, we measure the noise power density of the device in different temperatures, as shown in Figure 3f.

**Figure 4:**
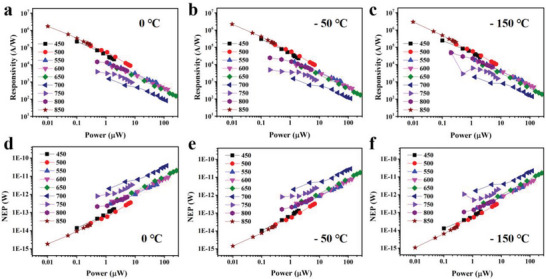
The responsivity of the device as a function of incident power at a) 0 °C, b) −50 °C, and c) −150 °C. The noise equivalent power (NEP) of the device as a function of incident power in d) 0 °C, e) −50 °C, and f) −150 °C.

**Figure** **4** displays the responsivity and noise equivalent power (NEP) of the device as a function of incident power in 0, −50, and −150 °C. We can conclude from Figure 4 that the responsivity of the device decreases linearly with the increase of incident light power, and NEP increases linearly with increasing incident light power. Figure S7 (Supporting Information) exhibits detection rate (D*) and photocurrent of the device with different wavelengths and incident light powers. The relationship between photocurrent and incident optical power is *I*_ph_ ∝ *P* ^*α*^, where *α* is the power factor of the device ranging from 0 to 1.^[^ ^39^ ^]^ The responsivity of the device is *R* = *I*_ph_/*P*, thus we can obtain the relationship *R* ∝ *P* ^−(1 − *α*)^ that the responsivity of the device will decrease with the increase of the incident power within a certain range.^[^ ^40^ ^]^ It can be seen from Figure 4a–c that the responsivity of the device increases with the decline of temperature (from −50 to −150 °C). The NEP of the device decreases with the decline of temperature, as shown in Figure 4d–f. Generally, there are localized energy levels in the bandgap of semiconductors for impurities and defects. The impurities of graphene/C_60_/Bi_2_Te_3_/C_60_/graphene heterojunction are mainly oxygen, water molecules, and the defects from the preparation process in films and graphene. After the impurities’ levels are filled, the nonequilibrium free carriers excited by the incident light will not be captured by the energy levels of impurities, which reduces the average life of the carriers and declines the photoconductive gain.^[^ ^41^, ^42^ ^]^ In a certain range, photoconductive gain is inversely proportional to the power, and the responsivity of the device will decrease with the increase of the incident power.

**Figure 5:**
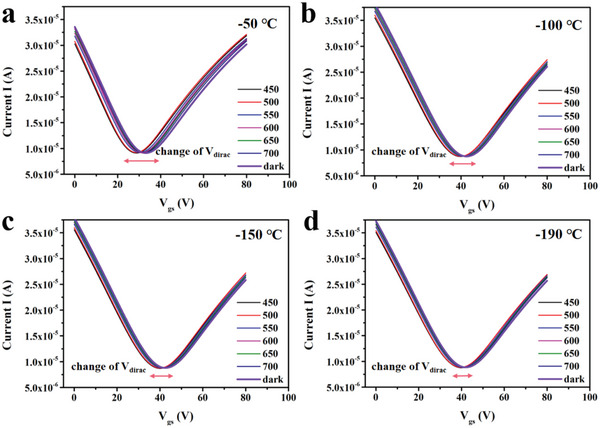
The transfer characteristic curve of the device at various wavelengths under a) −50 °C, b) −100 °C, c) −150 °C, and d) −190 °C.

We take silicon oxide as the dielectric layer of gate, and apply the gate voltage to obtain the transfer characteristic curve, as shown in **Figure** **5**. It can be seen that the device shows a good regulation ability of gate voltage, and the device clearly presents positive and negative response abilities as a function of gate voltage at different wavelengths. The Dirac point of intrinsic graphene is at the intersection of the top of the valence band and the bottom of the conduction band, and the corresponding Dirac voltage (*V*_Dirac_) is just at the zero point. If a positive gate voltage is applied to the device, its Fermi level will move to the valence band forming P doping and its *V*_Dirac_ will also move to the positive axis.^[^ ^43^ ^]^ If the gate voltage becomes negative, its Fermi level would move to the conduction band, forming N doping, and its *V*_Dirac_ will move to the negative axis. Figure 5 shows that *V*_Dirac_ of the device is on the positive axis, demonstrating that the graphene channel is P‐doped, and the dominant carriers are holes. Therefore, the doping type of graphene can be easily judged by its *I*_g_–*V*_g_ curve (transfer characteristic curve). After impurities and defects form localized energy levels in the bandgap, the conductivity strongly changes with temperature and its internal impurity concentration because the carrier concentration and Fermi level are determined by temperature and impurity concentration. With the temperature decreasing from −50 to −190 °C, the *V*_Dirac_ transfers from 25 to 40 V regardless of the light excitation, as shown in Figure S8 (Supporting Information). In this situation, the change of the Fermi level is only determined by temperature.

In addition, Figure 5a demonstrates that different incident lights will change the position of *V*_Dirac_, because the concentration of photoexcited charges from the impurity level was changed. Interestingly, the varying range of *V*_Dirac_ caused by different incident lights reduces as the temperature decreases. Keeping the incident light conditions unchanged, the varying range of *V*_Dirac_ caused by photoexcitation at −50 °C is significantly greater than the one at −190 °C. The reason can be attributed to the ionization degree of impurities, which is reduced with the decrease of temperature. It indicates that the position of the impurity energy level is close to the Fermi energy level as the temperature decreases, following Formulas (S1)–(S3) (Supporting Information).

We can infer from Figure 5a that the current is below the *I*_dark_ when *V*_g_ < *V*_Dirac_ and higher than *I*_dark_ when *V*_g_ > *V*_Dirac_ at −50 °C. In addition, same situation occurs at temperatures of −100, −150, and −190 °C. When the gate voltage is less than *V*_Dirac_, the graphene conductive channel is N‐type channel, and the conductive multicarriers are electrons. The injection of these nonequilibrium electrons will reduce the hole concentration, leading to the decrease of the device's current and a negative photocurrent response.

**Figure 6:**
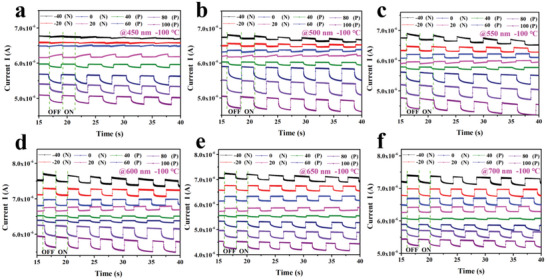
Response current at −100 °C under different gate voltages at a) 450, b) 500, c)550, d) 600, e) 650, and f)700 nm.

The electrical doping type of graphene can also be determined by giving a specific gate voltage, as shown in **Figure** **6**. The device has a negative response in the left range of the Dirac point, and a positive response in the right range. When the gate voltage is greater than *V*_Dirac_, the graphene conductive channel is a P‐type channel, and the conductive multicarriers are electrons.^[^ ^44^ ^]^ The complete process of photocurrent changes from negative to positive with an incident light at 450, 500, 550, 600, 650, and 700 nm, as shown in Figure 6 and Figure S9 (Supporting Information). As the gate voltage changes from −40 to 100 V, the photocurrent response gradually changes from negative to positive at −100 and −50 °C. When the incident light is in the visible light band, the composite films are mainly provided with photogenerated holes by the C_60_ and C_60_/graphene interfaces and injected into the graphene channels. When *V*_g_ is lower than 40 V, the conductive multicarriers are electrons in the graphene channel. The injection of the nonequilibrium holes would decrease the electron concentration and present a negative photocurrent response. Similarly, when *V*_g_ is higher than 40 V, the conductive multicarriers are holes in the graphene channel and the injection of the nonequilibrium holes would increase the hole concentration and present a positive photocurrent response.

Furthermore, to show the sensitivity and repeatability of the device, we give the photocurrent of the device when the minimum optical power is incident. As shown in Figure S10a (Supporting Information), our device exhibits good responses to weak optical power at 850 nm. Figure S10b,c (Supporting Information) shows the repeated *I*–*T* curve stability test with 680 nm light (switching frequency 1 Hz) after four months of the device's fabrication. To show the uniformity of the devices, we prepared 3 × 8 array graphene–C_60_–Bi_2_Te_3_–C_60_–graphene heterojunction phototransistors, and measured the dark‐current value and photocurrent value at a bias voltage 0.05 V (with 680 nm light), as shown in Figure S11 (Supporting Information). The coverage area of 3 × 8 array phototransistors is greater than 1 cm × 1 cm, which is enough to prepare more than 100 × 100 large‐scale array devices in the current lithography technology. To show the application potential of devices in array integration, we prepared 128 × 128 large‐scale integrated array devices and coupled with a readout circuit designed and prepared by us, as shown in Figures S12–S14 (Supporting Information).

## 3 Conclusion

In conclusion, we report a phototransistor with an ultrahigh responsivity and fast response time (3 × 10^6^ A W^−1^@0.25 ms). The graphene/C_60_/Bi_2_Te_3_/C_60_/graphene vertical heterojunction is placed on the source and drain electrodes forming a photoconductive transistor and patterned to micrometer size by DRIE (Deep Reactive Ion Etching) and photolithograph. At room temperature, the response band of the single device covers 400–1800 nm. The phototransistor not only exhibits a sensitive and fast response but also presents an adjustable bidirectional response, and we clearly explain the response mechanism. The thickness of the composite film is about 30 nm, which can realize a large area homogenization preparation and is compatible with planar lithography, etching, and nanoimprinting processes. Therefore, the phototransistor based on the composite film can be fabricated into large area array and combined with multifunctional nanostructures through nanofabrication methods like electron beam lithography and nanoimprinting, forming on‐chip spectrometer, polarization spectral imaging, and other functional array devices.

## 4 Experimental Section

### Photoresponse Characterization

Photoresponse characteristics of the heterojunction phototransistor were measured by a PDA(Platform Design Automation, Fs‐Pro 380) source meter analyzer and a supercontinuum laser source from 400 to 2400 nm (Anyang Company, Wuhan, China). All electrical tests were carried out under vacuum (vacuum degree was less than 5 × 10^−3^ Pa).

### AFM Measurements

AFM measurements were performed using an NT‐MDT AFM operating at room temperature.

### Raman and Optical Absorption Spectra

Raman spectra data were collected with a confocal microprobe Raman spectrometer (RENISHAW in Via Raman Microscope) with a 532 nm laser. The optical absorption spectra were measured by Lambda750 spectrophotometer (PerkinElmer).

### Statistical Analysis—Data Acquisition

The photocurrent was collected by taking the average of ten switching cycles within error 5%. The intensity of incident light spot was calculated according to uniform illumination when the size of light spot was less than 40 µm. The noise current conformed to the normal distribution and was read out by the noise analysis module in the semiconductor analyzer PDA. The numerical calculation was in strict accordance with the standard formula of photodetector. Responsivity: *R* = *I*_ph_/*P*; NEP: NEP = *P*/(*I*_ph_/*I*_noise_); D*: (*A*_d_Δ*f*)^1/2^ *I*_ph_/*I*_noise_/*P*.

### Statistical Analysis—Statistical Software

Graphs were prepared in Origin 9.0.

## Conflict of Interest

The authors declare no conflict of interest.

## Supporting information

Supporting Information

## Data Availability

The data that support the findings of this study are available from the corresponding author upon reasonable request.
