# Supplementary material for: High Performance Graphene–C60–Bismuth Telluride–C60–Graphene Nanometer Thin Film Phototransistor with Adjustable Positive and Negative Responses
Source: Adv Sci (Weinh). 2023 Feb 7;10(10):2206997. doi: 10.1002/advs.202206997 (PMC10074057; doi:10.1002/advs.202206997)
Supplement: Supplementary file 1 — Supporting Information [file ADVS-10-2206997-s001.pdf]

High Performance graphene-C_{60}-bismuth telluride-C_{60}-graphene nanometer thin film phototransistor with adjustable positive and negative responses

*Rui Pan, Yuanlingyun Cai, Feifei Zhang, Si Wang, Lianwei Chen, Xingdong Feng, Yingli Ha, Renyan Zhang, Mingbo Pu, Xiong Li, Xiaoliang Ma, and Xiangang Luo**

State Key Laboratory of Optical Technologies on Nano-Fabrication and Micro-Engineering, Institute of Optics and Electronics, Chinese Academy of Sciences, Chengdu 610209, China.
E-mail: lxg@ioe.ac.cn

R. Pan, Y. Cai, F. Zhang, S. Wang, L. Chen, X. Feng, Y. Ha, R. Zhang, M. Pu, X. Li, X. Ma, and X. Luo
State Key Laboratory of Optical Technologies on Nano-Fabrication and Micro-Engineering, Institute of Optics and Electronics, Chinese Academy of Sciences, Chengdu 610209, China.

L. Chen, Y. Ha, M. Pu
Research center on vector optical fields, Institute of Optics and Electronics, Chinese Academy of Sciences, Chengdu 610209, China

R. Pan, R. Zhang
Division of Frontier Science and Technology, Institute of Optics and Electronics, Chinese Academy of Sciences, Chengdu 610209, China

Y. Cai, X. Feng, Y. Ha, M. Pu, X. Li, X. Ma, X. Luo
School of Optoelectronics, University of Chinese Academy of Sciences, Beijing 100049, China.
Keywords: 2D material, graphene, phototransistor, bidirectional response, gate voltage regulation.

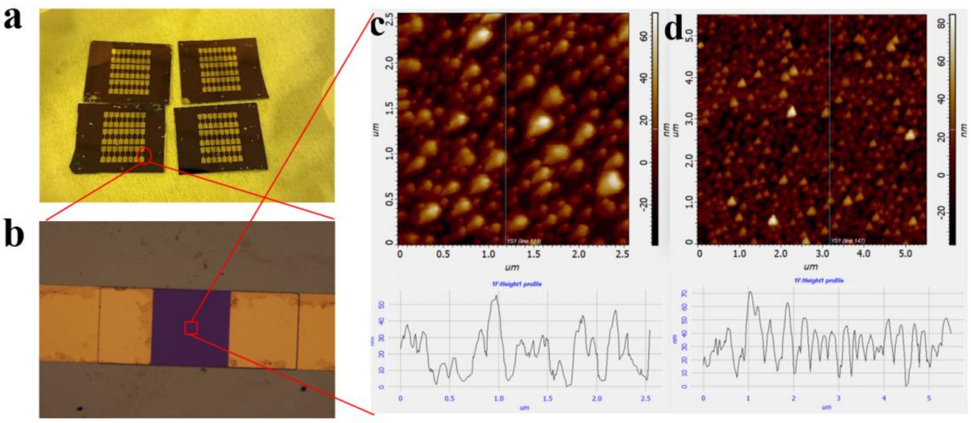

Figure S1 Optical images and AFM Characterization of devices. (a) The picture of the array devices. (b) Optical image of a single device. AFM characterization of the film for 2.5 μm (c) and 5 μm (d).

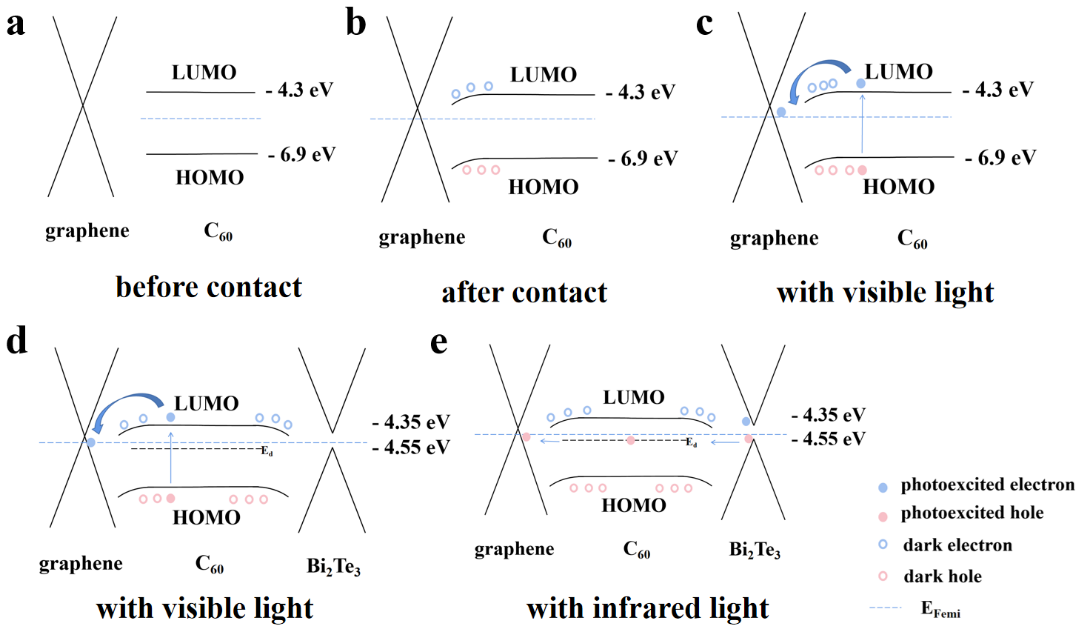

Figure S2 Energy band structure diagram of graphene/C_{60}/ Bi_2Te_3/C_{60}/graphene heterostructure.

Figure S2 exhibits the energy band structure of the heterojunction and its changes under visible and near-infrared light. After contact between graphene and C_{60}, a built-in barrier preventing hole injection into graphene forms due to the change of energy band, as shown in Figure S2b. After C_{60} contacts with Bi_2Te_3, a built-in barrier is generated to prevent electrons from entering C_{60}, as shown in Figure S2d.

Electron-holes pairs excited by visible light are transfered to the LOMO level from HOMO level of C_{60}, and the photogenerated electrons will transfer to the

graphene channel, as shown in Figures S2c and S2d. At this time, a negative photocurrent response is generated because the electrons are injected into the P-type doped graphene channel.

When the infrared light is illuminated, the photon energy is weak and it cannot excite the internal charge of C_{60}. However, electron-hole pairs can be generated in Bi_2Te_3, and the photogenerated holes enter the graphene channel through the impurity energy level of C_{60}. The photogenerated electrons cannot cross C_{60} to reach graphene due to the built-in potential barrier. The valence band top of Bi_2Te_3 is close to the impurity level in C_{60}, providing a hole transport channel. Therefore, photogenerated holes will enter the P-type graphene channel and generate a positive response under infrared light illumination, as shown in Figure S2e.

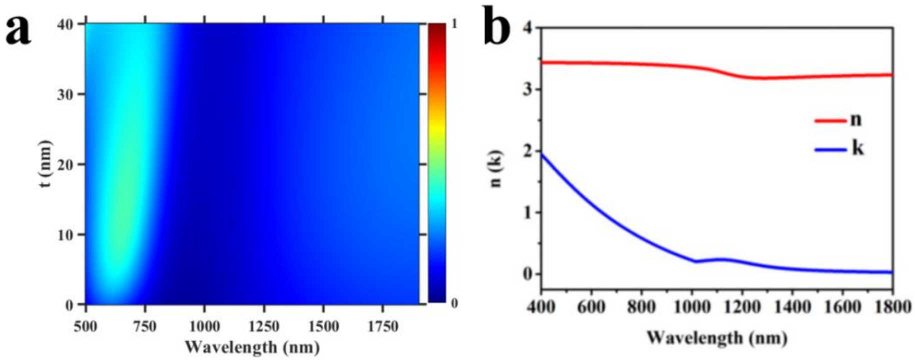

Figure S3 FDTD simulation results of Graphene/C_{60}/Bi_2Te_3/C_{60}/Graphene Films. (a) Absorption spectra of composite films with different thicknesses of Bi_2Te_3. (b) The real (n) and imaginary part (k) of refractive index of the grown Bi_2Te_3 film measured by ellipsometer.

Re: Thanks for the reviewer's suggestion. FDTD calculations were performed by the commercial software *Lumerical FDTD solutions*. Normal incident light from a plane wave source was imposed from the top of the multi-layer structure stacked in z direction, along with periodic boundary conditions utilized at x- and y-directions, and perfect matched layer at z direction. Specifically, graphene was modelled as a 1 nm thickness. The reflectance (R) and transmittance (T) were recorded during the calculation, then the absorption spectrum (A) was calculated by $A = 1 - R - T$. Refractive indexes of graphene and C_{60} were taken from the references [1,2], and the one of Bi_2Te_3 was experimentally obtained by ellipsometry (SENresearch SE850, the

results are shown in the Fig. S3 Supporting Information). The refractive indexes of Si and SiO_2 were taken from references[3].

[1] B. Song, H. Gu, S. Zhu, H. Jiang, X. Chen, C. Zhang, S. Liu, Appl. Surf. Sci. **2018**, 439, 1079.

[2] P. Eklund, A. Rao, Y. Wang, P. Zhou, K.-A. Wang, J. Holden, M. Dresselhaus, G. Dresselhaus, Thin solid films **1995**, 257, 211.

[3] Palik E. D, Handbook of Optical Constant of Solids. Academic Press, **1991**.

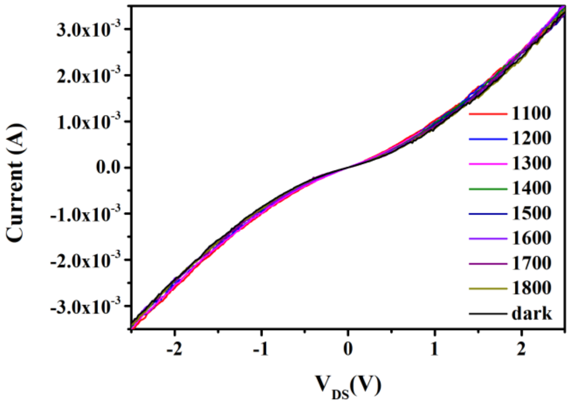

Figure S4 I-V curve as a function of wavelength in infrared region at room temperature

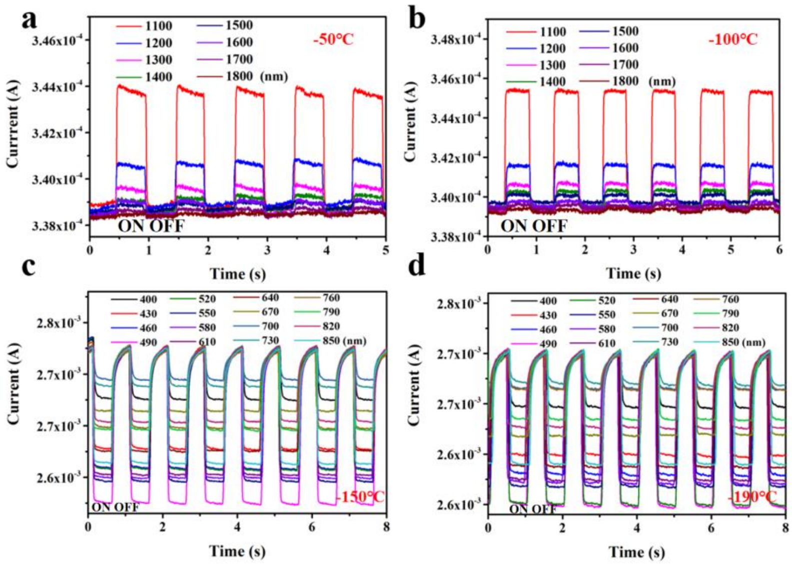

Figure S5 Photocurrent response of devices at 0 gate voltage under conditions: (a) at -50 ℃ in 1100-1800 nm, (b) at -100 ℃ in 1100-1800 nm, (c) at -150 ℃ in 400-850 nm, (d) at -190 ℃ in 400-850 nm.

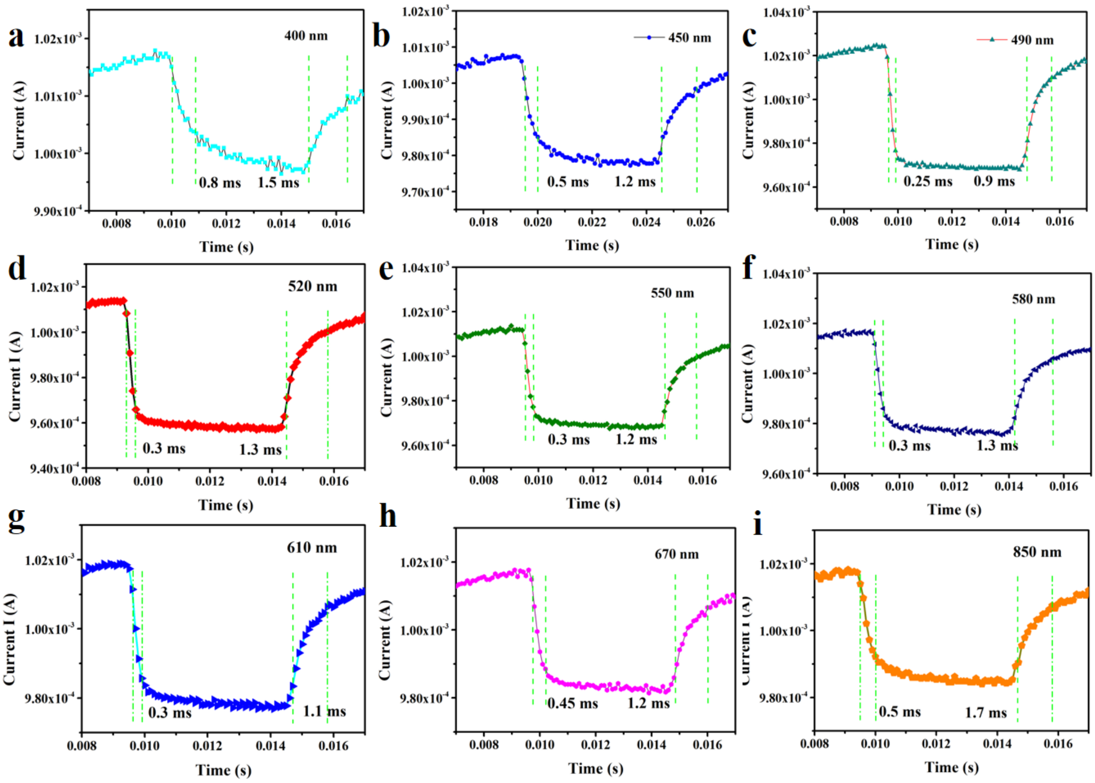

Figure S6 Response speed of devices under different wavelengths of incident light.

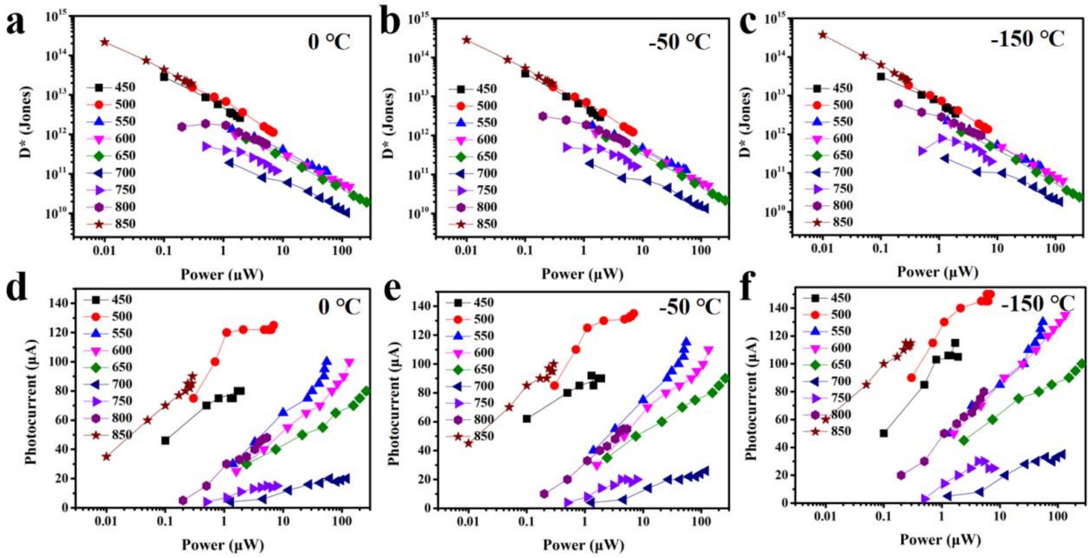

Figure S7 The respective detection rate (D*) and photocurrent of device as a function of wavelength and power of incident light at different temperatures: 0 ℃ (a, d), -50 ℃ (b, e), and -150 ℃ (c, f)

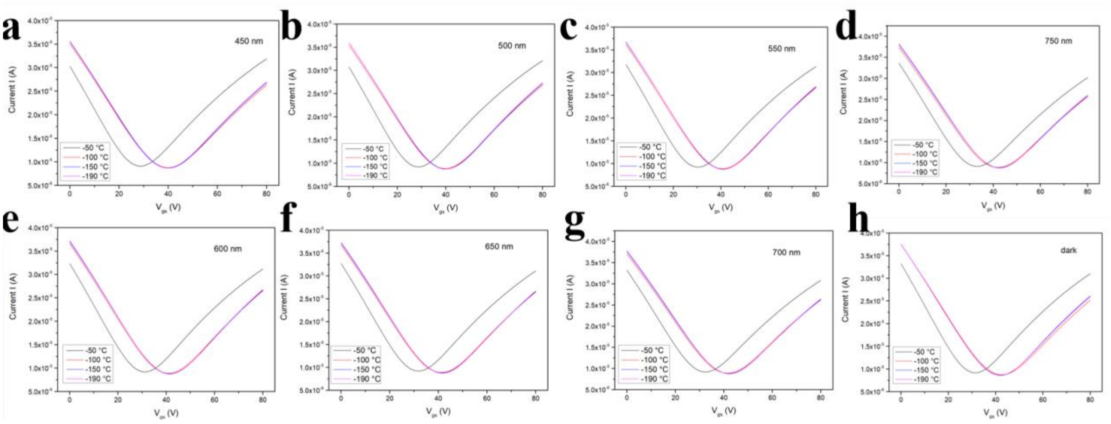

Figure S8 Transfer characteristic curves of devices at different temperatures and wavelengths.

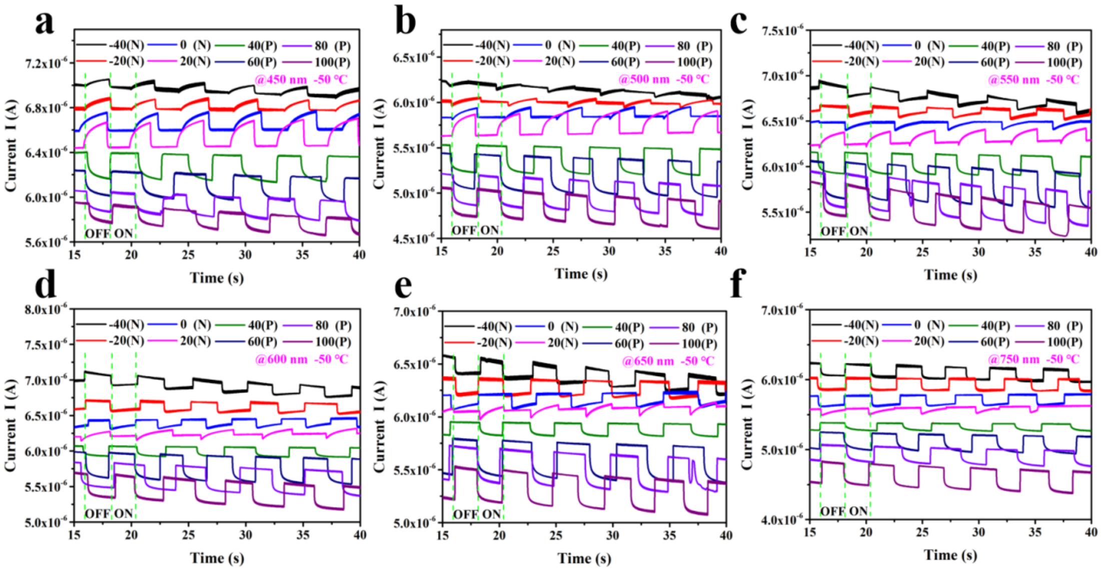

Figure S9 Response current at -50 ℃ under different gate voltages at (a) 450, (b) 500, (c)550, (d) 600, (e) 650, and (f)700 nm.

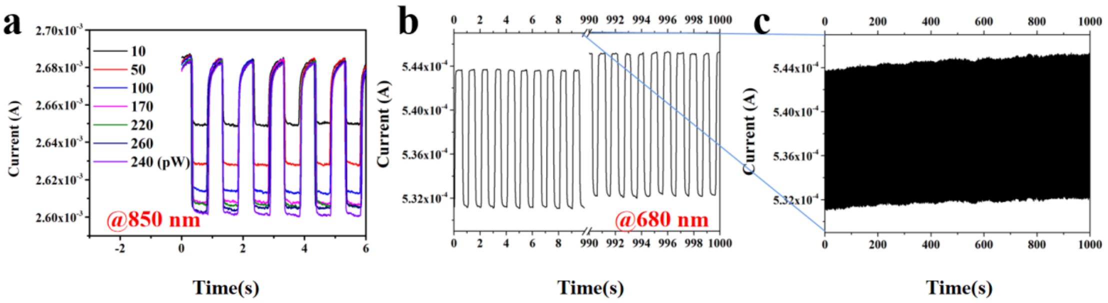

Figure S10 Sensitivity and repeatability test of the device. (a) The response current of the device under weak light intensity irradiation. (b)、(c) Repeated I-T curve stability test with 680 nm light after four months of fabrication.

a

I_{Dark} (A)<br>I_{photo}(μA)	Y1	Y2	Y3	Y4	Y5	Y6	Y7	Y8
X1	6.25×10^{-5}<br>0.15 μA	6.908×10^{-5}<br>0.2 μA	8.32×10^{-5}<br>0.2 μA	7.94×10^{-5}<br>0.2 μA	8.23×10^{-5}<br>0.21μA	8.718×10^{-5}<br>0.1μA	6.92×10^{-5}<br>0.07μA	8.236×10^{-5}<br>0.12μA
X2	5.785×10^{-5}<br>0.1 μA	6.245×10^{-5}<br>0.15 μA	4.752×10^{-5}<br>0.09 μA	6.65×10^{-5}<br>0.1 μA	6.902×10^{-5}<br>0.1 μA	5.386×10^{-5}<br>0.1 μA	6.102×10^{-5}<br>0.09 μA	5.315×10^{-5}<br>0.13 μA
X3	6.244×10^{-5}<br>0.12 μA	9.395×10^{-5}<br>0.15 μA	6.095×10^{-5}<br>0.2 μA	5.12×10^{-5}<br>0.1 μA	5.475×10^{-5}<br>0.13 μA	6.051×10^{-5}<br>0.07 μA	6.355×10^{-5}<br>0.11 μA	5.268×10^{-5}<br>0.06 μA

b

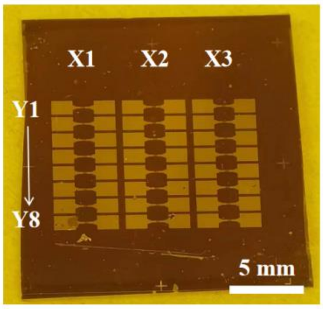

Figure S11 Dark-current and photo-current of 3 × 8 graphene-C_{60}- Bi_2Te_3-C_{60}-graphene heterojunction phototransistors array. The columns are marked by X1-3, while the rows are marked by Y1-8.

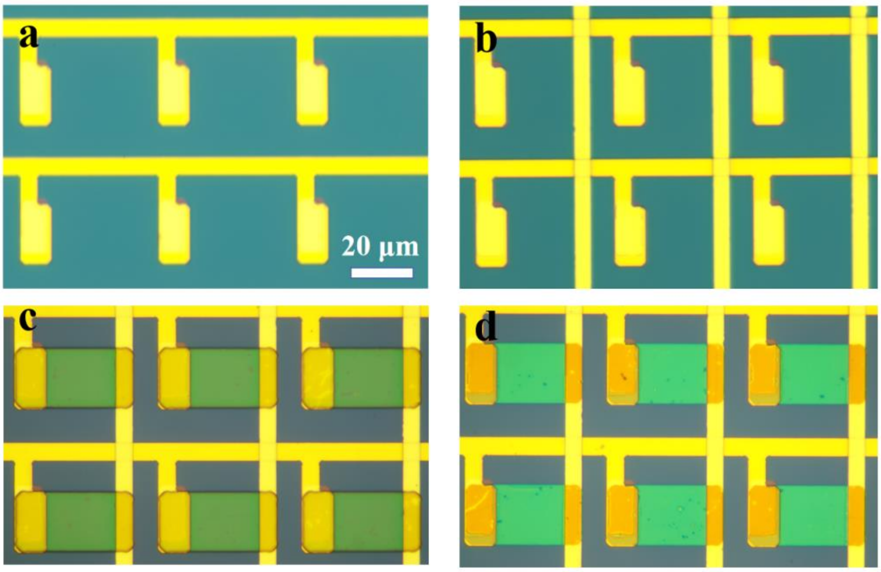

Figure S12 Preparation process of 128×128 graphene-C_{60}- Bi_2Te_3-C_{60}-graphene heterojunction phototransistor array device and readout circuit observed by optical microscope with 1000 times magnification.

Figures S12a and S12b exhibit the preparation process of readout circuit. Firstly, a layer of transverse metal electrode is prepared on the SiO_2/Si substrate. Then, a layer of silicon oxide (insulating layer) covers the transverse metal electrode, and a small

hole is left for connection, as shown in Figure S12a. After that, a longitudinal electrode is prepared on the silicon oxide insulating layer, as shown in Figure S12b. Finally, the heterojunction is fabricated into a suitable area to connect two layers of metal electrodes above and below the silicon oxide insulating layer by the preparation method described in the manuscript, as shown in Figure S12c (with photoresist) and Figure S9d (without photoresist). Figure S13 exhibits the same situation as Figure S12 but at a smaller magnification (500 times).

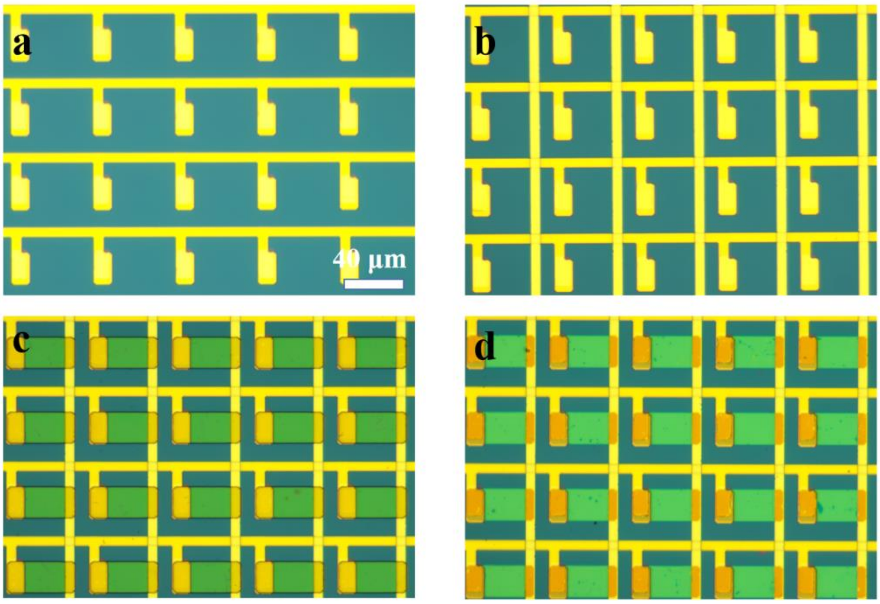

Figure S13 Preparation process of 128×128 graphene-C_{60}-Bi_2Te_3-C_{60}-graphene heterojunction phototransistor array device and readout circuit observed by optical microscope with 500 times magnification.

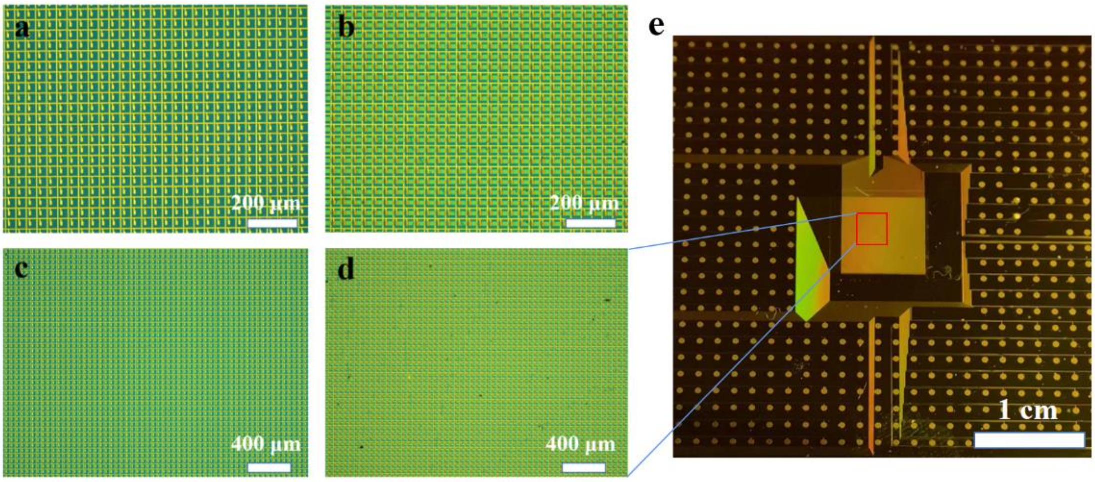

Figure S14 100 and 50 times magnification optical microscope diagram of 128×128 graphene-C_{60}- Bi_2Te_3-C_{60}-graphene heterojunction phototransistor array device and preparation process of readout circuit.

Figure S14a presents the 128 × 128 readout circuit, and Figure S14b presents graphene-C_{60}-Bi_2Te_3-C_{60}-graphene heterojunction phototransistor array device coupled with a readout circuit (Magnify 100 times). Figure S14c presents the 128 × 128 readout circuit, and Figure S14d presents graphene-C_{60}- Bi_2Te_3-C_{60}-graphene heterojunction phototransistor array device coupled with a readout circuit (Magnify 50 times). Figure S14e exhibits the overview of graphene-C_{60}-Bi_2Te_3-C_{60}-graphene heterojunction phototransistor array devices.

In general, the carriers mainly come from impurity ionization, while the intrinsic excitation is ignored. Only when the intrinsic carrier concentration does not exceed the temperature range of carrier concentration provided by impurity ionization, all impurities are ionized, so the carrier concentration is constant and the device work stably. E_F is discussed below according to different temperature ranges.

1. In weak ionization zone at the low temperature, most donor's energy levels of impurity are still occupied by electrons, while a small amount of impurity as donor ionize. , $n_0 = nD_+$.

$$\begin{cases} E_{\mathrm{F}} = \dfrac{E_{\mathrm{v}} + E_{\mathrm{A}}}{2} - \left(\dfrac{k_0 T}{2}\right) \ln\left(\dfrac{N_{\mathrm{A}}}{2N_{\mathrm{v}}}\right) \\ p_0 = \left(\dfrac{N_{\mathrm{A}} N_{\mathrm{v}}}{2}\right)^{\frac{1}{2}} \exp\left(-\dfrac{\Delta E_{\mathrm{A}}}{2k_0 T}\right) \end{cases} \quad \text{(S1)}$$

2. When the temperature rises to the point where most impurities are ionized, it is called strong ionization. $p_0 = N_A$, $P_A = D_+N_A$.

$$E_F = E_v - k_0T \ln \frac{N_A}{N_v}$$

$$D_+ = \left(\frac{2N_A}{N_v}\right)\exp\left(\frac{\Delta E_A}{k_0T}\right) \qquad \text{(S2)}$$

3. When the transition region is between the saturation region and the full intrinsic excitation, it is called the intrinsic excitation of the transition region. The electrons provided by the relative impurity ionization can no longer be ignored:

$$E_F = E_i - k_0Tsh^{-1}\left(\frac{N_A}{2n_i}\right) \quad p_0 = \left(\frac{N_A}{2}\right)\left[1+\left(1+\frac{4n_i^2}{N_A^2}\right)^{\frac{1}{2}}\right] \quad n_0 = \left(\frac{2n_i^2}{N_A}\right)\left[1+\left(1+\frac{4n_i^2}{N_A^2}\right)^{\frac{1}{2}}\right]^{-1} \qquad \text{(S3)}$$

For semiconductors with a specific impurity concentration, with the increase of temperature, the carrier transits from the impurity ionization as the primary source to the intrinsic excitation as the primary source, and E_F transits from the vicinity of the impurity level to the center line of the band gap. At a specific temperature, the position of Fermi energy level is determined by the type and concentration of impurities, and the position of Fermi energy level reflects the conductive type and doping level. Light incident at different wavelengths or powers will introduce photogenerated charges, which will change the carrier concentration, thus changing the Fermi energy level and the shift of the Dirac point. This is due to the change of Fermi energy level caused by the decrease of temperature.
